# Evaluation of pulmonary toxicity of benzalkonium chloride and triethylene glycol mixtures using *in vitro* and *in vivo* systems

**DOI:** 10.1002/tox.22722

**Published:** 2019-02-20

**Authors:** Doyoung Kwon, Yeon‐Mi Lim, Jung‐Taek Kwon, Ilseob Shim, Eunji Kim, Doo‐Hee Lee, Byung‐Il Yoon, Pilje Kim, Hyun‐Mi Kim

**Affiliations:** ^1^ Risk Assessment Division, Environmental Health Research Department National Institute of Environmental Research Incheon Republic of Korea; ^2^ Environmental Measurement & Analysis Center National Institute of Environmental Research Incheon Republic of Korea; ^3^ College of Veterinary Medicine Kangwon National University Chuncheon‐si Gangwon‐do Republic of Korea

**Keywords:** benzalkonium chloride, bronchoalveolar lavage fluid, inflammation, oxidative stress, pulmonary toxicity, triethylene glycol

## Abstract

Benzalkonium chloride (BAC) is a widely used disinfectant/preservative, and respiratory exposure to this compound has been reported to be highly toxic. Spray‐form household products have been known to contain BAC together with triethylene glycol (TEG) in their solutions. The purpose of this study was to estimate the toxicity of BAC and TEG mixtures to pulmonary organs using *in vitro* and *in vivo* experiments. Human alveolar epithelial (A549) cells incubated with BAC (1‐10 μg/mL) for 24 hours showed significant cytotoxicity, while TEG (up to 1000 μg/mL) did not affect cell viability. However, TEG in combination with BAC aggravated cell damage and inhibited colony formation as compared to BAC alone. TEG also exacerbated BAC‐promoted production of reactive oxygen species (ROS) and reduction of glutathione (GSH) level in A549 cells. However, pretreatment of the cells with *N*‐acetylcysteine (NAC) alleviated the cytotoxicity, indicating oxidative stress could be a mechanism of the toxicity. Quantification of intracellular BAC by LC/MS/MS showed that cellular distribution/absorption of BAC was enhanced in A549 cells when it was exposed together with TEG. Intratracheal instillation of BAC (400 μg/kg) in rats was toxic to the pulmonary tissues while that of TEG (up to 1000 μg/kg) did not show any harmful effect. A combination of nontoxic doses of BAC (200 μg/kg) and TEG (1000 μg/kg) promoted significant lung injury in rats, as shown by increased protein content and lactate dehydrogenase (LDH) activity in bronchoalveolar lavage fluids (BALF). Moreover, BAC/TEG mixture recruited inflammatory cells, polymorphonuclear leukocytes (PMNs), in terminal bronchioles and elevated cytokine levels, tumor necrosis factor α (TNF‐α), and interleukin 6 (IL‐6) in BALF. These results suggest that TEG can potentiate BAC‐induced pulmonary toxicity and inflammation, and thus respiratory exposure to the air mist from spray‐form products containing this chemical combination is potentially harmful to humans.

**Abbreviations**
ANOVA: analysis of variance
BAC: benzalkonium chloride
BALF: bronchoalveolar lavage fluids
BCA: bicinchoninic acid
DCFDA: dichlorofluorescin diacetate
DDAC: didecyldimethylammonium chloride
DTNB: 5,5′‐dithiobis‐2‐nitrobenzoic acid
EG: ethylene glycol
GSH: glutathione
GSSG: oxidized glutathione
H&E: hematoxylin and eosin
HV: heptylviologen
IC_50_: inhibitory concentration 50
IL‐6: interleukin 6
IS: internal standard
LC_50_: lethal concentration 50
LD_50_: lethal dose 50
LDH: lactate dehydrogenase
MRM: multiple reaction‐monitoring
MTT: 3‐(4,5‐dimethylthiazol‐2‐yl)‐2,5‐diphenyltetrazolium bromide
NAC: *N*‐acetylcysteine
PBS: phosphate buffered saline
PMNs: polymorphonuclear leukocytes
QAC: quaternary ammonium compounds
ROS: reactive oxygen species
TEG: triethylene glycol
TNF‐α: tumor necrosis factor α

## 1 INTRODUCTION

Benzalkonium chloride (BAC) is a widely used cationic surfactant belonging to a group of compounds known as quaternary ammonium compounds (QAC). BAC is a mixture of *n*‐alkylbenzyldimethylammonium chlorides, with various alkyl (R) chain lengths from C_8_H_17_ to C_18_H_37_.^1^ BACs with an alkyl chain of *n* = 10‐16 are known to have potent bactericidal effects, and are commonly used as disinfectants, antiseptics, and preservatives.^2^ Many studies have shown the harmful effects of BAC on the human body, such as skin irritation and allergic contact dermatitis.^3^, ^4^, ^5^ Highly toxic effects of BAC have also been found in animal studies. BAC orally administered to rats causes significant lethality, and the lethal dose 50 (LD_50_) has been reported to be 234‐525 mg/kg.^6^, ^7^ Pulmonary effects of BAC have particularly been investigated since this preservative has been used in inhalable medications. BAC included in nebulized bronchodilators for treatment of asthma and chronic obstructive pulmonary diseases has been reported to cause bronchoconstriction and bronchospasm.^8^, ^9^, ^10^ Rodents that singly or repeatedly inhaled BAC show pulmonary irritation, inflammation, and damage to the blood‐air barrier.^11^, ^12^, ^13^ Moreover, BAC administered via oral and intravascular routes in rats causes pulmonary edema and interstitial pneumonia, with a higher tissue concentration of BAC in the lungs than in the blood and other organs. Thus, it has been suggested that lung can be a reservoir of BAC and consequently a target organ for BAC toxicity.^14^, ^15^

Triethylene glycol (TEG) is a liquid used as a solvent, a humectant, and a chemical intermediate.^16^ TEG mist or vapor has been used for disinfection because it shows bactericidal activity against pneumococci, streptococci, and staphylococci.^17^, ^18^, ^19^ TEG is relatively safe for humans, and very low toxicities have been shown in animals administered with TEG via oral, intravenous, and percutaneous routes.^16^ The acute oral LD_50_ of TEG is reported to be 22 000 mg/kg in rats^20^ and more than 18 500 mg/kg in mice.^16^ A rodent inhalation test showed that acute respiratory exposure to TEG aerosol is not toxic.^21^ However, humans repeatedly exposed to aerosol containing TEG experience coughing, shortness of breath, wheezing, and chest tightness,^16^ indicating that chronic inhalation of TEG is potentially harmful to the respiratory organs.

In comparison to the toxicities of individual substances, those of chemical mixtures are quite unpredictable owing to the possibility of toxic interactions between the chemicals, which can induce synergism, addition, potentiation, or antagonism of their effects on biological systems.^22^, ^23^, ^24^ Some commercial spray‐form household products such as sanitizers and deodorants include BAC (0.01%‐1.8%, w/v) and TEG (1%‐15%, w/v) as disinfectants and/or preservatives in their solutions.^25^ Since the air mist particles from these products are inhalable, human respiratory organs can be directly exposed to BAC and TEG. Nonetheless, the pulmonary effects of BAC and TEG in combination have not been reported. The present study was designed to estimate the combined toxicity of BAC and TEG to lung cells and tissues. Human alveolar epithelial (A549) cells and rat pulmonary organs were exposed to BAC and TEG either individually or together, and the cellular and clinical responses were observed. The results showed that the BAC and TEG mixture caused more severe pulmonary injury than the individual compounds alone.

## 2 MATERIALS AND METHODS

### 2.1 Chemicals

All chemicals including BAC and TEG were purchased from Sigma‐Aldrich (St. Louis, MO).

### 2.2 Cell culture

Human adenocarcinomic alveolar epithelial (A549) cells were obtained from the Korea Cell Bank (Seoul, Korea). Cells were grown in RPMI 1640 medium (Thermo Fisher Scientific Inc., Waltham, MA) containing 10% heat‐inactivated fetal bovine serum (Thermo Fisher Scientific Inc.) and 1% penicillin‐streptomycin (Thermo Fisher Scientific Inc.) under standard cell culture conditions (37°C, 5% CO_2_, and 90% humidity).

### 2.3 Determination of in vitro cytotoxicity

The concentrations of BAC used in *in vitro* toxicity tests were decided based on blood and/or tissue concentrations of BAC in previous studies. The concentrations of BAC in the whole blood and lung were 0.06‐0.34 μg/g and 0.39‐2.75 μg/g, respectively, in rats after oral administration of BAC 250 mg/kg.^14^, ^15^ The serum BAC level of a human individual who accidentally swallowed BAC was 0.16 μg/mL several hours after ingestion.^26^ The dose of TEG was determined based on the concentration ratios of BAC and TEG in household products.^25^ For the single chemical toxicity test, A549 cells (5 × 10^4^ cells) were incubated in 96‐well plates overnight, and were treated with BAC (0.3, 1, 2, 3, 4, 6, 8, and 10 μg/mL) or with TEG (0.3, 1, 3, 10, 30, 100, 300, and 1000 μg/mL). To evaluate the combined toxicity, 2 μg/mL of BAC (around its IC_10_ of 1.63 μg/mL) and various concentrations of TEG (0, 1, 3, 10, 30, 100, 300, and 1000 μg/mL) were added to the cell culture medium. After 24 hours incubation, cell viability was determined by a 3‐(4,5‐dimethylthiazol‐2‐yl)‐2,5‐diphenyltetrazolium bromide (MTT) assay showing the mitochondrial/cytosolic reducing capacity of NAD(P)H oxidoreductase, which converts soluble MTT reagent to insoluble formazan in living cells.^27^ Cells were incubated with 200 μg/mL of MTT in culture medium for 2 hours and then the medium was removed. After washing with phosphate‐buffered saline (PBS), dimethyl sulfoxide was added to the well. After gentle shaking, the absorbance was measured at 540 nm by microplate reader. The activity of LDH in the culture media released from the cells through the damaged membrane was quantified after 24 hours exposure to the chemicals using a QuantiChrom LDH Kit (BioAssay Systems, Hayward, CA). The activity of LDH from totally disrupted cells treated with PBS containing 1% triton X‐100 for 1 hour was considered 100%, and the relative activity of LDH of each group was calculated.

### 2.4 Colony formation assay

Clonogenic assay was conducted using the method of Franken et al.^28^ Cells were incubated in 6‐well plates at the density of 250 cells per well overnight. BAC (0.3 μg/mL) with or without TEG (1, 10, 100, or 1000 μg/mL) was incubated with the cells for 10 days. After incubation, the medium was discarded, and the cells were washed with 70% ethanol followed by staining with 0.5% crystal violet in 20% methanol solution. The numbers and the sizes of colonies were quantified using light microscopy (Olympus Co., Tokyo, Japan).

### 2.5 Determination of cellular oxidative stress

Intracellular generation of ROS was determined using dichlorofluorescein diacetate (DCFDA). A549 cells in black‐well plates were treated with the test materials for 24 hours and then incubated with PBS containing 25 μM DCFDA for 30 minutes. After washing with PBS, the fluorescence was measured at λ_exitation_ 499 nm and λ_emission_ 522 nm using a microplate reader. Total GSH level was determined using the 5,5′‐dithiobis‐2‐nitrobenzoic acid (DTNB)‐GSH reductase recycling method.^29^ Cells exposed to BAC with or without TEG for 24 hours were harvested and homogenized in PBS containing 5 mM EDTA and 0.1% Triton X‐100. Centrifuged (10 000*g*, 4°C, 10 minutes) supernatant was incubated with the mixture of DTNB, NADPH, and GSH reductase, and the change of absorbance was monitored at 412 nm for 1 minute. Oxidized GSH (GSSG) was determined using 2‐vinlypyridine, which was added to the same samples prior to the assay. The protein content of cell lysate was measured by a Bicinchoninic Acid (BCA) Protein Assay Kit (iNtRON Biotechnology, Seoul, Korea). NAC (1 mM) was treated onto the cells 1 hour before the exposure of BAC and TEG mixture, and MTT assay was conducted after 24 hours incubation.

### 2.6 Determination of BAC concentration using LC/MS/MS

A549 cells (1 × 10^7^ cells) were exposed to BAC (2 μg/mL) with or without TEG (1, 10, 100, or 1000 μg/mL) for 24 hours. Cells were harvested and homogenized with PBS containing 0.1% Triton X‐100 and then centrifuged (10 000*g*, 4°C, 5 minutes). The supernatant was mixed with an equivalent volume of methanol and injected into a high‐performance liquid chromatography (HPLC) system (Surveyor MS Pump and Auto Sampler, Thermo Fisher Scientific Inc., Waltham, MA) equipped with a Kinetex 2.6 μm, C_18_, 100 × 2.1 mm column (Phenomenex Inc., Torrance, CA). The column temperature was 35°C. An aqueous solution containing 10 mM ammonium acetate and 0.1% acetic acid was used as mobile phase A, and methanol containing 10 mM ammonium acetate and 0.1% acetic acid was used as mobile phase B. The concentration gradient was changed as follows at a flow rate of 0.5 mL/min: Solvent A at 0‐1 minute 70%, 3‐4 minutes 0%, and 5‐8 minutes 70%. The HPLC system was connected to a TSQ quantum triple quadrupole mass spectrometer (Thermo Fisher Scientific Inc.) and equipped with an electrospray ionization probe operated in the positive ionization mode. The peaks were detected in multiple reaction monitoring (MRM) mode. Four major components of BAC with different alkyl chain lengths BAC‐C_10_, ‐C_12_, ‐C_14_, and ‐C_16_, were determined, and the retention times were 2.98, 3.25, 3.50, and 3.73 minutes, respectively. The sum of four major components was regarded as total BAC, and heptylviologen (HV) was used as an internal standard (IS). Detailed conditions of MRM for BAC and HV analysis are summarized in Table 1. The absorption of BAC by the cells was determined by the calculation of ratio between the intracellular amount of BAC and the total amount of BAC added to the culture media.

**Table 1:** LC/MS/MS MRM conditions for BAC analysis

Compound	Precursor ion (m/z)	Product ion (m/z)	Cone voltage (V)	Collision energy (eV)
BAC‐C_10_	276.3	184.1 > 91.1	102	18, 34
BAC‐C_12_	304.3	212.2 > 91.1	89	21, 34
BAC‐C_14_	332.3	240.2 > 91.1	96	22, 32
BAC‐C_16_	360.3	268.2 > 91.1	96	21, 31
Heptylviologen (IS)	382.5	255.2 > 157.1	92	22, 37

### 2.7 Animals and treatments

Male Sprague‐Dawley rats (7 weeks old) were purchased from Orient Bio Inc. (Seongnam, Korea). Rats were acclimated to temperature (22 ± 3°C) and humidity (50 ± 10%) controlled rooms with 12 hours light/dark cycle for 1 week before treatment. The animal experiment protocol was approved by the Institutional Animal Care and Use Committee of the National Institute of Environmental Research (NIER‐15‐1). TEG and BAC, dissolved in PBS (pH 7.4), were intratracheally instilled into the rats, and the injection volume was 1 mL/kg body weight. For testing the pulmonary effect of individual chemicals, PBS, BAC (100, 200, and 400 μg/kg), or TEG (100, 500, and 1000 μg/kg) were administered to rats (five rats per group), and the BALF were obtained at 1 day after exposure. The concentrations of BAC and TEG in the injected solution were 0.01‐0.04% (w/v) and 0.01‐0.1% (w/v), respectively, considering the usual concentration of BAC (0.01‐1.8%) in household spray products and the concentration ratios of BAC and TEG in the products. To identify the combined toxicity of the two chemicals, 60 rats (10 rats per group) were treated with PBS, TEG (1000 μg/kg), BAC (100 or 200 μg/kg), and the mixtures (TEG 1000 μg/kg + BAC 100 μg/kg, or TEG 1000 μg/kg + BAC 200 μg/kg). The rats were sacrificed at 1 day (6 rats per group) and 7 days (4 rats per group) postexposure, and the BALF and lung tissues were collected.

### 2.8 BALF analysis

Rats were intraperitoneally injected with 50 mg/kg of tiletamine + zolazepam (Zoletil 50, Virbac; Carros, France) and 15 mg/kg of xylazine hydrochloride (Rumpun, Bayer; Leverkusen, Germany) for anesthetization. BALF was collected via five lavages of the lung with a total of 25 mL PBS (pH 7.4, magnesium‐ and calcium‐free) for analysis of pulmonary toxicity and inflammation. The BALF were centrifuged (200*g*, 4°C, 10 minutes) and the cell‐free supernatant of the first lavage was used for determination of total protein content, LDH activity, TNF‐α, and IL‐6 levels. Total protein content was determined by a BCA assay kit (iNtRON Biotechnology), and LDH activity was determined using a QuantiChrom LDH Kit (BioAssay Systems). TNF‐α and IL‐6 levels were quantified using Quantikine enzyme‐linked immunosorbent assay kits (R&D Systems; Minneapolis, MN). Centrifuged cell pellets in all lavages were re‐suspended in PBS, and total cells were counted using a Vi‐Cell XR analyzer (Beckman Coulter; Brea, CA). The cells were fixed on slide glass via centrifugation (1800 rpm, 4 minutes) using a Shadon Cytospin 4 Cytocentrifuge (Thermo Fisher Scientific Inc.) and stained with Diff‐Quick (Sysmex Corporation, Kobe, Japan). Macrophages and PMNs were counted using light microscopy (Olympus Co.; Tokyo, Japan).

### 2.9 Histopathological examination

Lung tissues were fixed in 10% neutral buffered formalin and embedded in paraffin. The tissues were sectioned at 3‐5 μm thickness and mounted on slide glass. The slides were stained with hematoxylin and eosin (H&E), and a histopathological examination was performed using dual microscopes (Olympus Co., Tokyo, Japan).

### 2.10 Statistical analysis

All results were expressed as the mean ± SE. Means of different groups were compared using two‐tailed unpaired Student's *t* tests or one‐way analysis of variance (ANOVA, Tukey's multiple comparison test) by GraphPad Prism version 5.0 software (GraphPad Software Inc., La Jolla, CA).

## 3 RESULTS

### 3.1 Cytotoxicity induced by BAC and TEG in A549 cells

**Figure 1:**
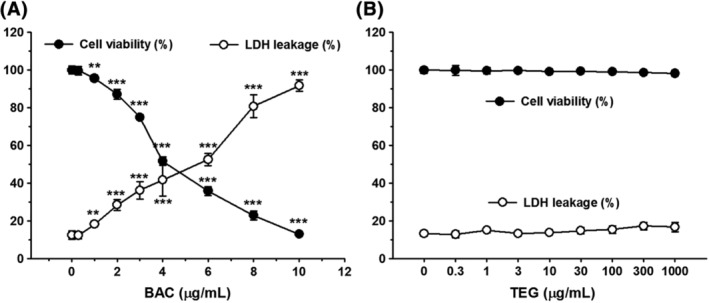
Cytotoxicity of BAC and TEG to human alveolar epithelial (A549) Cells. A, Cytotoxicity of BAC. B, Cytotoxicity of TEG. A549 cells were treated with BAC or TEG for 24 hours. Cell viability and membrane damage were determined by MTT and LDH assays, respectively. Mean ± SE. Student's *t* test, ^**,***^ *P* < 0.01 and 0.001, respectively, vs corresponding control

**Figure 2:**
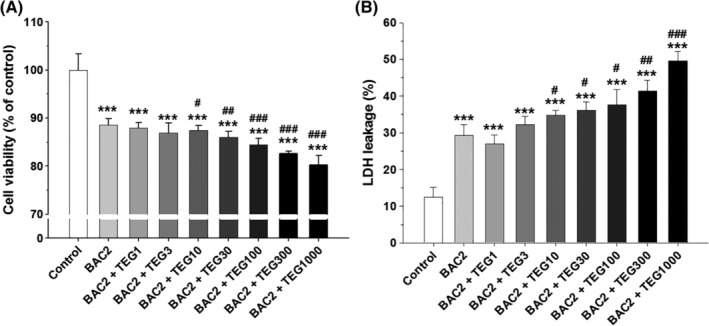
Cytotoxicity induced by the combinations of BAC and TEG in human alveolar epithelial (A549) cells. A, Cell viability (MTT). B, Cell membrane damage (LDH). A549 cells were treated with BAC (2 μg/mL) with or without TEG (1‐1000 μg/mL) for 24 hours. Mean ± SE. Student's *t* test, ^***^ *P* < 0.001 vs control. ^#,##,###^ *P* < 0.05, 0.01, and 0.001, respectively, vs BAC alone

Human alveolar cells treated with BAC (1‐10 μg/mL) for 24 hours showed a dramatic decrease in cell viability and increase in cell membrane damage as determined by MTT and LDH assays, respectively (Figure 1A). Most of the cells were dead by 10 μg/mL of BAC (Figure 1A) much lower than its concentrations (0.1‐18 mg/mL) in commercial products. The IC_10_ value of BAC calculated from the results of the MTT assay was 1.63. The IC_50_ of BAC (5.04 μg/mL, approximately 14.8 μM as a major form of BAC with alkyl chain C_12_H_25_) in the present study is in the similar range of previously reported IC_50_ value of BAC (22.8 μM) in osteosarcoma cybrid cells.^30^ TEG did not have any adverse effect on the cells at any concentrations (0.1‐1000 μg/mL) tested (Figure 1B). However, combined exposure to TEG (10‐1000 μg/mL) and BAC (2 μg/mL) significantly decreased cell viabilities (Figure 2A) and increased LDH leakages (Figure 2B) as compared to BAC alone in a TEG‐dose‐dependent manner.

### 3.2 Inhibition of colony formation of A549 cells induced by BAC and TEG

**Figure 3:**
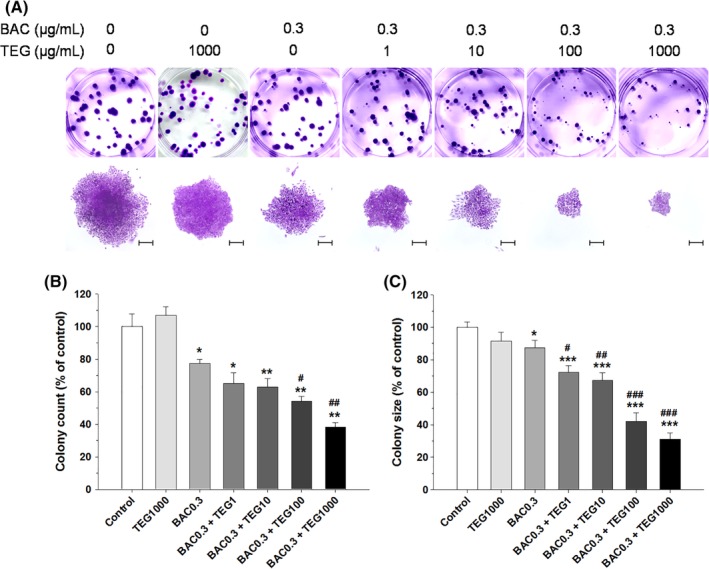
Colony formation of human alveolar (A549) cells treated with BAC and/or TEG. A, Culture plate and microscopic images of cell colonies. B, Colony number. C, Colony size. A549 cells cultured at low density (250 cells/well) were exposed to BAC (0.3 μg/mL) with or without TEG (1‐1000 μg/mL) for 10 days. Mean ± SE. Student's *t* test, ^*,**,***^ *P* < 0.05, 0.01, and 0.001, respectively, vs control. ^#,##,###^ *P* < 0.05, 0.01, and 0.001, respectively, vs BAC alone. Scale bar = 200 μm [Color figure can be viewed at wileyonlinelibrary.com]

TEG (up to 1000 μg/mL) exposure to the alveolar cells cultured at low density (250 cells/well) for 10 days did not affect colony formation (Figure 3). However, BAC significantly inhibited cell proliferation in a dose‐dependent manner, and more than 1 μg/mL of BAC eliminated almost all cells (data not shown). Thus, a lower concentration of BAC (0.3 μg/mL) was mixed with various concentrations of TEG (1‐1000 μg/mL) and treated onto the cells. BAC alone decreased the colony number (23%) and colony size (13%) as compared to the nontreated control. The addition of TEG to BAC resulted in a dramatic decrease in both colony number and size (Figure 3). These results indicate that a combination of BAC and TEG has much greater inhibitory effect on alveolar cell replication and survival as compared to the individual chemicals.

### 3.3 Oxidative stress induced by BAC and TEG in A549 cells

**Figure 4:**
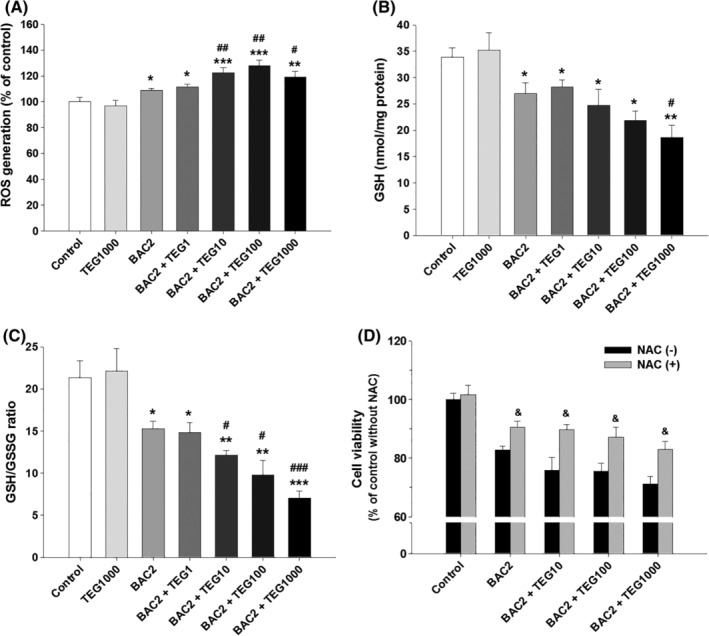
Oxidative stress induced by the mixtures of BAC and TEG in human alveolar (A549) cells. A, ROS generation. B, Cellular total GSH level. C, GSH/GSSG ratio. D, Cell viability. A549 cells were exposed to BAC (2 μg/mL) with or without TEG (1‐1000 μg/mL) for 24 hours. NAC (1 mM) was treated onto the cells 1 hour before exposure to BAC and TEG. Mean ± SE. Student's *t* test, ^*,**,***^ *P* < 0.05, 0.01, and 0.001, respectively, vs control. ^#,##,###^ *P* < 0.05, 0.01, and 0.001, respectively, vs BAC alone. ^&^ *P* < 0.05 vs corresponding group without NAC

To identify the mechanism of toxicity induced by the two chemicals, cellular oxidative stress was determined, since BAC has been reported to be an ROS‐producing agent in human cells.^31^, ^32^, ^33^ BAC (2 μg/mL) increased ROS production by 8.7% as compared to the control group, while TEG alone (up to 1000 μg/mL) did not affect ROS generation (Figure 4A). However, 10 and 100 μg/mL of TEG in combination with BAC (2 μg/mL) elevated ROS production to 22.3% and 28.0%, respectively, as compared to the control (Figure 4A). In cells treated with BAC and the highest concentration of TEG (1000 μg/mL), only a 19% increase in ROS production was detected, probably due to the decrease of cell viability in this group. The intracellular concentration of GSH was not changed by TEG alone but was reduced by BAC (2 μg/mL) by about 20% (Figure 4B). The addition of 1, 10, and 100 μg/mL TEG to BAC did not make a statistical difference in the total GSH concentration, but 1000 μg/mL of TEG with BAC reduced the GSH level significantly as compared to BAC alone. An indicator of cellular redox state, the ratio of GSH and GSSG, was also significantly reduced by BAC, and was decreased more by the BAC and TEG (10‐1000 μg/mL) mixture (Figure 4C). Pretreatment with NAC, a metabolic precursor of GSH, significantly alleviated cytotoxicity induced by BAC and TEG, indicating that oxidative stress can be one of the mechanisms of cell damage (Figure 4D).

### 3.4 Intracellular absorption of BAC in the presence of TEG in A549 cells

**Figure 5:**
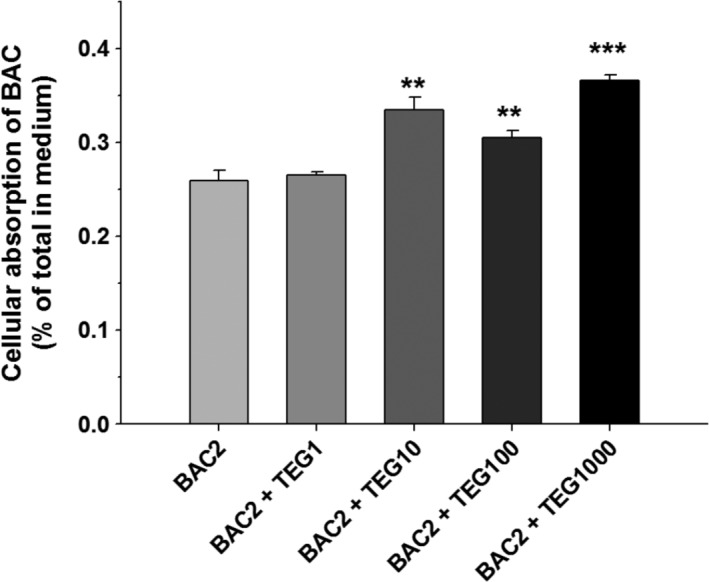
Cellular absorption of BAC in the presence of TEG. A549 cells were exposed to BAC (2 μg/mL) with or without TEG (1‐1000 μg/mL) for 24 hours, and the intracellular BAC was quantified by LC/MS/MS. Mean ± SE. Student's *t* test, ^**,***^ *P* < 0.01, and 0.001, respectively, vs BAC alone

Previously, we reported that organic solvent ethylene glycol (EG)increased the cellular distribution of didecyldimethylammonium chloride (DDAC), which also has a structure of QAC, and potentiated the toxicity of DDAC to human bronchial cells.^34^ In the present study, we hypothesized that the cellular absorption of BAC could be changed by TEG, and thus the intracellular BAC content was measured using the LC/MS/MS system. Although the same amount of BAC (2 μg/mL) was treated onto all groups, cells incubated with BAC and TEG together showed a higher intracellular BAC concentration as compared to the cells treated with BAC alone (Figure 5). Thus, it can be suggested that the enhancement of BAC absorption into cells by TEG could be a reason for the increased toxicity.

### 3.5 Pulmonary toxicity induced by intratracheally instilled BAC and TEG in rats

**Figure 6:**
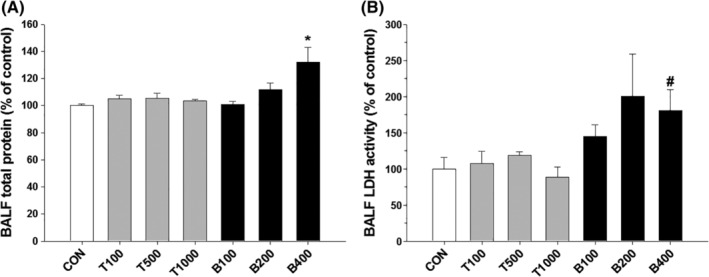
Acute pulmonary toxicity induced by BAC or TEG in rats. A, Total protein content in BALF. B, LDH activity in BALF. Male rats were intratracheally instilled with BAC (B; 100, 200, or 400 μg/kg) or TEG (T; 100, 500, or 1000 μg/kg), and the toxicity was determined using BALF at 1 day after exposure. Mean ± SE. Student's *t* test, ^*^ *P* < 0.05 vs control, ^#^ *P* = 0.06 vs control

**Figure 7:**
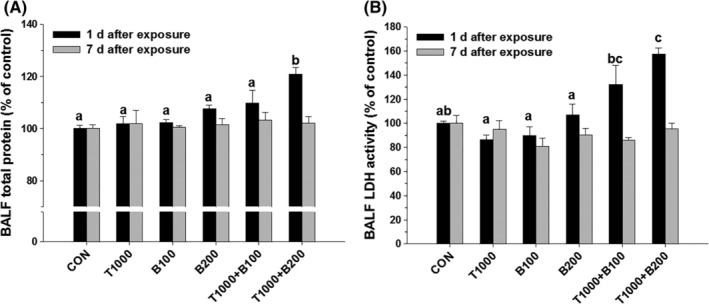
Pulmonary toxicity induced by a mixture of BAC and TEG in rats. A, Total protein content in BALF. B, LDH activity in BALF. Male rats were intratracheally instilled with BAC (B; 100 or 200 μg/kg) and TEG (T; 1000 μg/kg), individually or together. BALF was obtained from the rats after 1 or 7 days exposure. Mean ± SE. Values with different letters (a, b, c) are significantly different from one another, and the letter indicates the comparison among the groups at 1 day after exposure. Mean ± SE. One‐way ANOVA followed by Tukey's multiple comparison test

To evaluate the toxic effects of BAC and TEG mixtures on animal pulmonary organs, rats were directly injected with BAC and TEG individually or together intratracheally. Acutely injected 100 and 200 μg/kg of BAC did not change total protein contents or LDH activities in the BALF, but 400 μg/kg of BAC significantly increased these two biomarkers of pulmonary cell damage (Figure 6) at 1 day posttreatment. In contrast, TEG (100, 500, and 1000 μg/kg) did not show any pulmonary toxicity (Figure 6). To identify the influence of the BAC and TEG combination, TEG (1000 μg/kg) was mixed with a sub‐toxic dose of BAC (100 or 200 μg/kg) and administered to rats. Significantly elevated protein contents (Figure 7A) and LDH activities (Figure 7B) were found at 1 day in the higher dose of BAC (200 μg/kg) mixture group but not in the lower dose of BAC (100 μg/kg) mixture group. At 7 days after exposure, the toxic effects of the chemical mixtures were normalized to the control level, indicating recovery from the pulmonary injury. No differences in body and lung weight were observed between the groups (data not shown).

### 3.6 Pulmonary inflammation induced by intratracheally instilled BAC and TEG in rats

**Figure 8:**
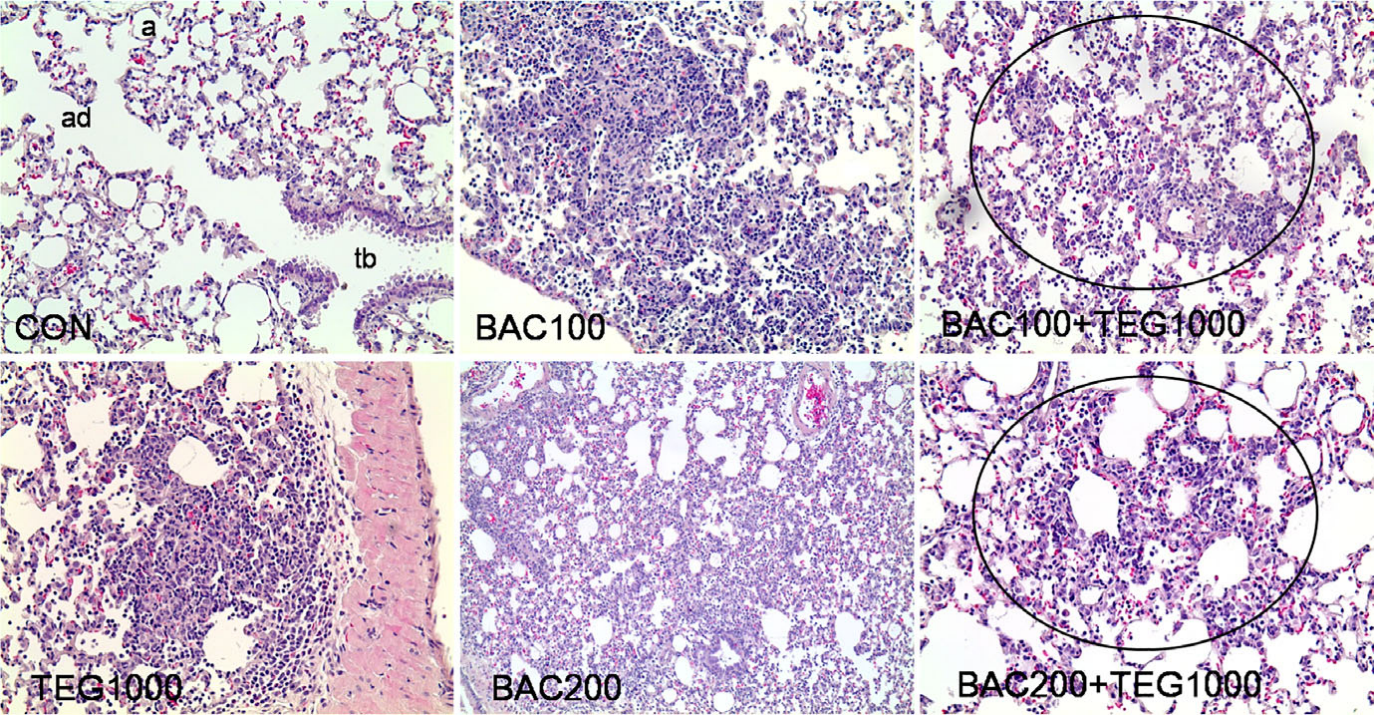
Histopathology of lung tissues from rats intratracheally exposed to BAC and TEG. Male rats were intratracheally instilled with BAC (100 or 200 μg/kg) and TEG (1000 μg/kg), either individually or together. Lung tissues were isolated and stained with H&E after 1 day exposure. Multifocal bronchiolar/alveolar acute inflammation in the circle. tb, terminal bronchioles; ad, alveolar ducts; a, alveoli. H&E. Magnification, 100× for BAC200 and 200× for others [Color figure can be viewed at wileyonlinelibrary.com]

**Figure 9:**
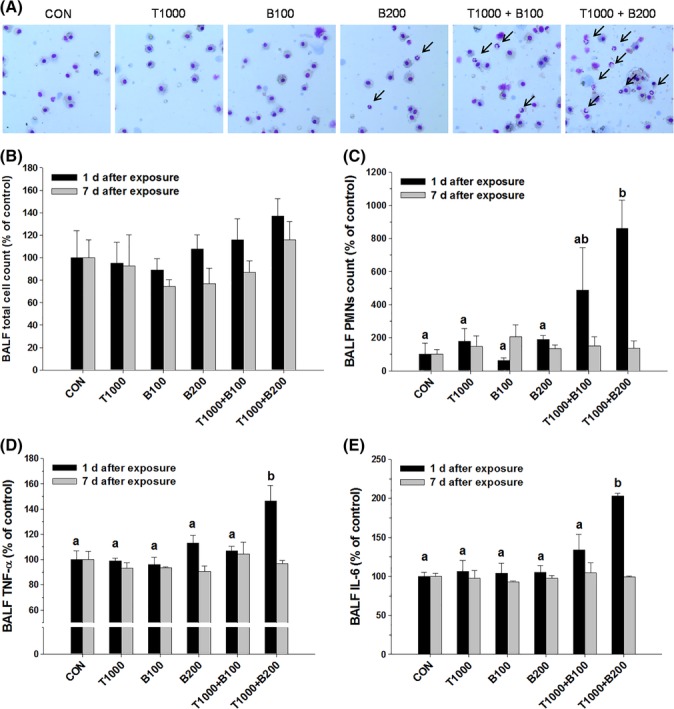
Pulmonary inflammation induced by BAC and TEG combinations in rats. A, Diff‐quick staining of BALF cells. B, Total cell count in BALF. C, PMNs in BALF. D, TNF‐α in BALF. E, IL‐6 in BALF. Male rats were intratracheally instilled with BAC (B; 100 or 200 μg/kg) and TEG (T; 1000 μg/kg), individually or together. BALF was obtained from the rats after 1 or 7 days exposure. Arrow indicates PMNs. Mean ± SE. Values with different letters (a, b) are significantly different from one another, and the letter indicates the comparison among the groups at 1 day after exposure. One‐way ANOVA followed by Tukey's multiple comparison test [Color figure can be viewed at wileyonlinelibrary.com]

In the H&E stained lung tissues, no significant differences between control group and single chemical‐treated groups were found. However, in the BAC and TEG mixture groups, infiltration of inflammatory cells, PMNs, was observed in terminal bronchioles (Figure 8). Microscopic images of BALF cells showed similar numbers of total cells in all groups (Figure 9A,B), but greatly increased levels of PMNs were shown only in the mixture (BAC 200 μg/kg and TEG 1000 μg/kg)‐treated rats at 1 day after exposure (Figure 9A,C). Inflammatory cytokines, TNF‐α and IL‐6, were also increased when both BAC (200 μg/kg) and TEG were injected into the rat lungs, while individual compounds did not alter their levels (Figure 9D,E). These changes in inflammatory cytokines showed similar patterns to the PMN recruitment between the groups, suggesting a close association between the inflammatory cells and cytokine secretion. At 7 days after exposure, the inflammatory responses to the BAC and TEG mixture were reduced and returned to the control level.

## 4 DISCUSSION

The QAC are a chemical category of cationic surfactants that have a common structure of NR_4_^+^ with alkyl or aryl groups of R.^35^, ^36^ Their potent bactericidal activities have been attributed to their aliphatic long alkyl chains that permeate into the cell membrane lipid bilayers. The charged nitrogen that remains at the surface of the membrane disturbs the charge distribution, finally resulting in the destruction of cell membrane structure and the leakage of intracellular molecules.^35^, ^36^, ^37^ Not only microorganisms but also human pulmonary cells can be subjected to the toxicity of QAC including BAC. An in vitro study using human normal bronchial epithelial (BEAS‐2B) cells showed that 2 hours incubation of 0.005 (50 μg/mL) to 0.01 (100 μg/mL) % BAC significantly damages cell membranes and decreases the cell viability.^38^ Pulmonary exposure to BAC in rats has also been reported to be highly toxic, with a lethal concentration 50 (LC_50_) of acute (4 hours) and repeated (3 days, 6 hours/day) inhalations of 53 and 30 mg/m^3^, respectively.^12^ In the present study, BAC also showed significant toxicity by a membrane‐destructive mechanism in human alveolar epithelial cells as determined by LDH released in the culture media (Figure 1A). The IC_50_ of BAC obtained from A549 cells in the present study, 5.04 μg/mL, is relatively lower than the previously reported IC_50_ in BEAS‐2B, about 35 μg/mL (0.0035%).^38^ However, this gap of BAC cytotoxicity in A549 and BEAS‐2B cells could be due to the different incubation time, 24 and 2 hours^38^, respectively. Although quite different susceptibilities to inhalable toxicants have been reported between A549 and BEAS‐2B cells,^39^, ^40^, ^41^ there has been no comparative study about the toxic effects of BAC on these two pulmonary cell lines. The *in vivo* results indicate that an intratracheal injection of BAC 400 μg/kg is toxic to rat lung tissues as evidenced by the increases of BALF protein and LDH levels (Figure 6). LDH and total proteins, the biomarkers of pulmonary toxicity, are leaked into bronchoalveolar space from injured bronchial/alveolar cells and damaged airway structures.^42^, ^43^ It has been also shown that inhalation exposures to BAC cause significant increases of BALF protein and LDH in mice^11^ and rats.^12^, ^13^ In contrast to BAC, the pulmonary toxicity of TEG is known to be very low; the LC_50_ of acute TEG aerosol inhalation by rats is greater than 4400 mg/m^3^.^16^ In the present study, TEG did not show any harmful effects on cultured alveolar cells at concentrations up to 1000 μg/mL (0.1%, Figure 1B) or on rat lung at doses up to 1000 μg/kg (Figures 6 and 7).

Compared to the single compounds, the BAC and TEG combination led to more potent pulmonary toxicity in our *in vitro and in vivo* systems. The increased toxicity of BAC by TEG in the alveolar cells was clearly shown not only by MTT and LDH assays (Figure 2) but also by clonogenic assay (Figure 3). Clonogenic assay is an *in vitro* technique for evaluating the effect of a certain agent on cell survival and proliferation via observation of the number and size of cell colonies after exposure of single cells to the agent.^28^ This assay can be used in cancer research to estimate the anti‐tumorigenic activities of drug candidates and also in toxicological research to identify the adverse effects of chemicals.^44^, ^45^ In the present study, TEG itself did not affect the colony formation, but remarkably inhibited A549 cell survival and proliferation in combination with BAC (Figure 3).

The results of animal experiments also show that the combination of BAC and TEG can promote unpredictable pulmonary toxicity. We acutely exposed rats to a sub‐toxic dose of BAC (100 or 200 μg/kg) together with a nontoxic dose of TEG (1000 μg/kg) via intratracheal route. However, intriguingly, the combination (BAC 200 μg/kg and TEG 1000 μg/kg) caused critical lung injuries along with increases in LDH and total proteins in BALF 1 day after treatment. Thus, it appears that BAC (200 μg/kg) alone was insufficient to damage the lung tissues, but TEG could enhance the membrane‐disrupting action of BAC on the rat bronchoalveolar epithelial cells and airway structures, which was similarly observed in our *in vitro* data. The concentration of BAC 0.02% (w/v, 200 μg/mL) in the intratracheally injected solution, which caused lung toxicity in combination with TEG, is within the concentration range (0.01%‐1.8%, w/v) of BAC^25^ in commercial spray‐form household products. Therefore, it can be suggested that even though the products contain individually safe concentrations of BAC and TEG, the aerosol of the mixed solution can have adverse effects on human respiratory organs.

BAC and TEG mixture administration also provoked lung inflammatory responses that were not observed in rats treated with each individual compound (Figures 8 and 9). Pulmonary inflammation is usually implicated in lung injuries caused by inhaled substances.^46^, ^47^ When respiratory spaces are exposed to exogenous materials, inflammatory responses are initiated by the activation of pulmonary macrophages that remove the substances via phagocytosis, and various inflammatory mediators are secreted by the cells to attract other immune cells such as PMNs.^46^, ^47^ PMNs, including neutrophils, basophils, and eosinophils, migrate to the damaged and inflamed sites from the blood circulation to clear the foreign substances.^46^, ^47^ PMNs are observed predominantly in bronchoalveolar regions at the early stage of pulmonary inflammation, whereas macrophages are the major cell type in those regions in healthy animals.^48^ Thus, the number of total cells and the percentage of PMNs in BALF can be useful indicators of pulmonary inflammatory responses.^46^ In the present study, only the BAC and TEG mixture injection induced local inflammation, as shown by the infiltration of PMNs in terminal bronchioles, while TEG or BAC alone did not affect lung tissue histology (Figure 8). Inflammatory cytokines are known to mediate the initiation and maintenance of pulmonary inflammation.^49^, ^50^ TNF‐α, mainly produced by activated alveolar macrophages, promotes the adherence of circulating inflammatory cells to the lung endothelium and stimulates the secretion of other inflammatory mediators such as IL‐6.^51^, ^52^ IL‐6 acts as a chemoattractant to recruit other inflammatory cells such as PMNs, which can also be sources of these cytokines.^53^, ^54^, ^55^, ^56^ Therefore, the BALF concentrations of TNF‐α and IL‐6 are highly increased in acute pulmonary inflammation.^45^, ^57^ In the present study, elevated BALF TNF‐α and IL‐6 levels in mixture‐administered rats (TEG 1000 μg/kg and BAC 200 μg/kg) were closely associated with the increased PMN recruitment. Taken together, the BAC and TEG mixture induced inflammatory cell attraction to pulmonary tissues via cytokine secretion, probably by activated macrophages. Since TNF‐α also has a cytotoxic effect on cells,^52^ acutely induced inflammation might be involved in the toxicity caused by the mixture.

Previously, it was reported that the combination of DDAC, another biocide belonging to the QAC group, and several organic surfactants showed synergistic antimicrobial effects. DDAC at a sub‐lethal concentration exerts bactericidal activity against *Staphylococcus aureus* in the presence of nonionic surfactants such as polyoxypropylene glycol, polyoxyethylene lauryl ether, and polyoxyethylene isodecyl ether.^58^ In that study, it was suggested that nonionic surfactants facilitate the penetration of DDAC into the bacterial membrane. Recently, we reported the combined toxicity of DDAC and EG to pulmonary cells. DDAC‐induced cytotoxicity is potentiated in the presence of nontoxic doses of EG in human bronchial epithelial (BEAS‐2B) cells, and the effect is dependent on the concentration of EG.^34^ Moreover, rats intratracheally instilled with the mixture of DDAC and EG show significant pulmonary toxicity and inflammation, whereas the individual chemicals do not induce toxicity in respiratory organs.^59^ These findings appear to be quite similar to the results of the present study, with the same aspects of cytotoxicity induced by the combination of the QAC and organic solvent. In the previous report, the enhancement of cellular absorption of DDAC facilitated by EG was suggested to be a possible mechanism of toxicity because elevated concentrations of intracellular DDAC were detected in cells treated with both DDAC and EG as compared to cells exposed to DDAC alone.^34^ Similarly, in the present study, TEG treatment enhanced the intracellular distribution of BAC in alveolar epithelial cells. The surfactant and/or emulsifying action of the BAC and TEG combination to the cell membrane structure could be a possible reason for the increased penetration of BAC, but the exact mechanism of the altered membrane permeability is unclear.

Along with its effects on cell membrane structure, the influence of BAC on intracellular biomolecules, organelles, and their functions is another possible mechanism of the increased toxicity resulting from higher absorption of BAC into cells by TEG. BAC impairs mitochondrial ATP synthesis and O_2_ consumption by direct inhibition of mitochondrial complex I in human corneal epithelial cells.^30^ Many studies have shown that oxidative stress induced by BAC is important to its toxicity to human cell lines. Incubation of BAC with cultured human corneal epithelial cells results in dose‐dependent increases in ROS production and DNA damage.^33^ Significant production of ROS with BAC treatment has also been observed in a human keratinocyte (HaCaT) cell line, and NADPH oxidase is suggested to be the source of the ROS.^32^ In human conjunctival epithelial cells, BAC decreases cell membrane integrity, induces chromatin condensation, and promotes excessive formation of reactive oxidants such as H_2_O_2_ and superoxide anions.^31^ Oxidative stress is usually involved in the inhalable toxicant‐associated pathogenesis of human lung diseases.^60^, ^61^ The interactions between oxidative free radicals and cellular macromolecules, such as proteins, DNA, and lipids, lead to impairment of cell functions and finally cell death.^60^, ^61^ GSH is a major cellular antioxidant that protects biomolecules against reactive oxidants by its reducing capacity and is converted to GSSG.^61^ In the present study, BAC induced significant oxidative stress in alveolar cells (Figure 5). TEG alone did not alter the ROS production, GSH content, or GSH/GSSG ratio (data not shown), but in combination with BAC aggravated cellular oxidative stress more than BAC alone (Figure 5). Alleviation of mixture‐induced cell death by pretreatment with NAC strongly supports that the oxidative stress is an important mechanism of cytotoxicity of the mixture. Peroxidation of membrane lipid by ROS perturbs the bilayer structure and increases membrane permeability.^60^, ^61^ Therefore, the oxidative stress caused by the BAC and TEG mixture in the present results might be also associated with membrane damage in the cells.

In conclusion, BAC and TEG combinations induced toxicity in pulmonary cells and tissues as compared to either chemical alone. The toxic effects of BAC on human alveolar cells, including ROS overproduction, membrane destruction, and cell death, were potentiated in the presence of TEG. The enhancement of cellular BAC absorption by TEG appears to be the mechanism of toxicity. In rats, a sub‐toxic dose of BAC instilled into the trachea induced significant pulmonary injury and inflammation in combination with a nontoxic dose of TEG. These results show that TEG can amplify the toxic effects of BAC on animal pulmonary organs. Although each compound in spray‐form household products is considered safe, pulmonary exposure to BAC and TEG together can be potentially harmful to human health. To examine the exact effect of this chemical combination on the respiratory system, further rodent inhalation experiments are currently underway in our lab.

## CONFLICT OF INTEREST

The authors declare that there is no conflict of interest.
